# Targeted inhibition of glutaminase as a potential new approach for the treatment of *NF1* associated soft tissue malignancies

**DOI:** 10.18632/oncotarget.21573

**Published:** 2017-10-06

**Authors:** Tahir N. Sheikh, Parag P. Patwardhan, Serge Cremers, Gary K. Schwartz

**Affiliations:** ^1^ Herbert Irving Comprehensive Cancer Center, New York, NY, USA; ^2^ Department of Pathology and Cell Biology, College of Physicians and Surgeons, Columbia University, New York, NY, USA; ^3^ Department of Hematology/Oncology, Columbia University College of Medicine, New York, NY, USA

**Keywords:** NF1, glutaminase, CB-839, glutamine, soft-tissue sarcoma

## Abstract

Many cancer cells rely on glutamine as the source of carbon molecules to feed the biosynthetic pathways and are often addicted to glutaminolysis. Inhibitors of glutaminase activity have gained attention in the last few years due to their anti-proliferative effect and ability to induce apoptosis in some cancers. Although it is a promising therapeutic approach, its efficacy or the role played by glutamine in modulating cell proliferation in *NF1* associated tumors has never been studied. We report for the first time, a strong correlation between the *NF1* status of tumor cells and increased sensitivity to glutamine deprivation and glutaminase inhibition. Soft-tissue cell lines null for *NF1* were highly dependent on glutamine for proliferation and showed decreased mTORC1 and Ras activity in response to glutaminase inhibition. Re-addition of glutamine or intermediary metabolite such as glutamate to the media restored mTORC1 and Ras activity. SiRNA mediated *NF1* knockdown in wild-type *NF1* cell line shows increased sensitivity to glutaminase inhibition. Conversely, *NF1* overexpression in *NF1* null cell lines results in reduced sensitivity to glutaminase inhibition, and restores mTORC1 signaling and Ras activity. These findings provide new insights into the role played by glutamine metabolism in *NF1* associated tumors and strongly warrant further investigation as a potential therapy in the *NF1* disease setting.

## INTRODUCTION

Normal cells produce energy mostly through the oxidation of pyruvate in the mitochondria; however, cancer cells are known to produce energy via increased glycolysis in the cytosol. This effect known as “Warburg Effect” [1] requires a metabolic shift from oxidative phosphorylation to glycolysis or lactate fermentation [2]. Glutamine, one of the most abundant intracellular amino acids, plays an important role in satisfying the biosynthetic needs of proliferating cancer cells by providing carbons to produce tricarboxylic acid (TCA) cycle intermediates, glutathione, fatty acids, and nucleotides [3–6]. As a result of glutamine being a major source of carbon molecules in tumor growth-facilitating metabolic pathways, many cancer cells often become “addicted” to glutaminolysis (a rate limiting step in the TCA cycle) [5]. Glutaminolysis occurs via two steps, first step is catalyzed by glutaminase (GLS) and converts glutamine to glutamate. The second step converts glutamate to α-ketoglutarate (α-KG) and is catalyzed by glutamate dehydrogenase (GDH) [7]. Cancer cells addicted to glutaminolysis often rely on glutamine as the carbon source for the TCA cycle [8].

In humans, GLS exists in two forms: kidney-type glutaminase (GLS1) and liver-type glutaminase (GLS2). While GLS1 is expressed ubiquitously, GLS2 is expressed primarily in the liver [9]. Recent efforts have focused on targeting glutaminolysis by inhibiting the GLS activity in cancer cells [10]. Drugs targeting GLS activity such as BPTES (bis-2-(5-phenylacetamido-1,2,4-thiadiazol-2-yl)ethyl sulfide 3) or CB-839 have gained attention owing to their potent inhibition of GLS1 activity and anti-proliferative effect in multiple tumor subtypes including leukemia and triple negative breast cancer [11].

Neurofibromatosis type 1 (NF1) is an autosomal dominant genetic disorder caused due to the loss and/or mutation of *NF1* tumor suppressor gene [12]. The *NF1* gene codes for a Ras GTPase activating protein called Neurofibromin (NF) and mutational inactivation and/or loss of *NF1* can lead to altered Ras-MAPK signaling [13]. Many patients with NF1 are often at risk of developing cancers such as gliomas, neurofibromas and malignant peripheral nerve sheath tumors (MPNSTs) among others [14, 15]. MPNSTs are soft-tissue tumors that are highly aggressive with a very poor prognosis [16]. *NF1* associated MPNSTs are often fatal and there are not many treatment options available to treat these therapeutically resistant tumors.

Although glutamine metabolism has been shown to play a crucial role in tumorigenesis both *in vitro* and *in vivo* [17], its role in *NF1* disease setting has not been studied before. In this study, we report for the first time that *NF1* associated soft-tissue sarcoma cell lines (MPNST, ST8814, S462) are highly dependent on glutamine for proliferation compared to wild-type *NF1* cell lines (LS141, CHP100, STS26T). Targeted inhibition of glutaminase (GLS) using inhibitors BPTES and CB-839 results in significant inhibition of cell proliferation and mTORC1 activity. Association between glutamine metabolism and *NF1* was also confirmed using siRNA and *NF1* over-expression studies *in vitro*. Furthermore, treatment of MPNST xenografts with CB-839 resulted in significant suppression of tumor volume and inhibition of downstream signaling pathways. Results obtained in this study strongly suggest that glutaminase inhibition in *NF1* associated tumors needs to be explored for a potentially novel therapeutic approach in this disease setting.

## RESULTS

### *NF1* mutant/null cell lines show decreased cell viability and mTORC1 activity in response to glutamine deprivation

**Figure 1:**
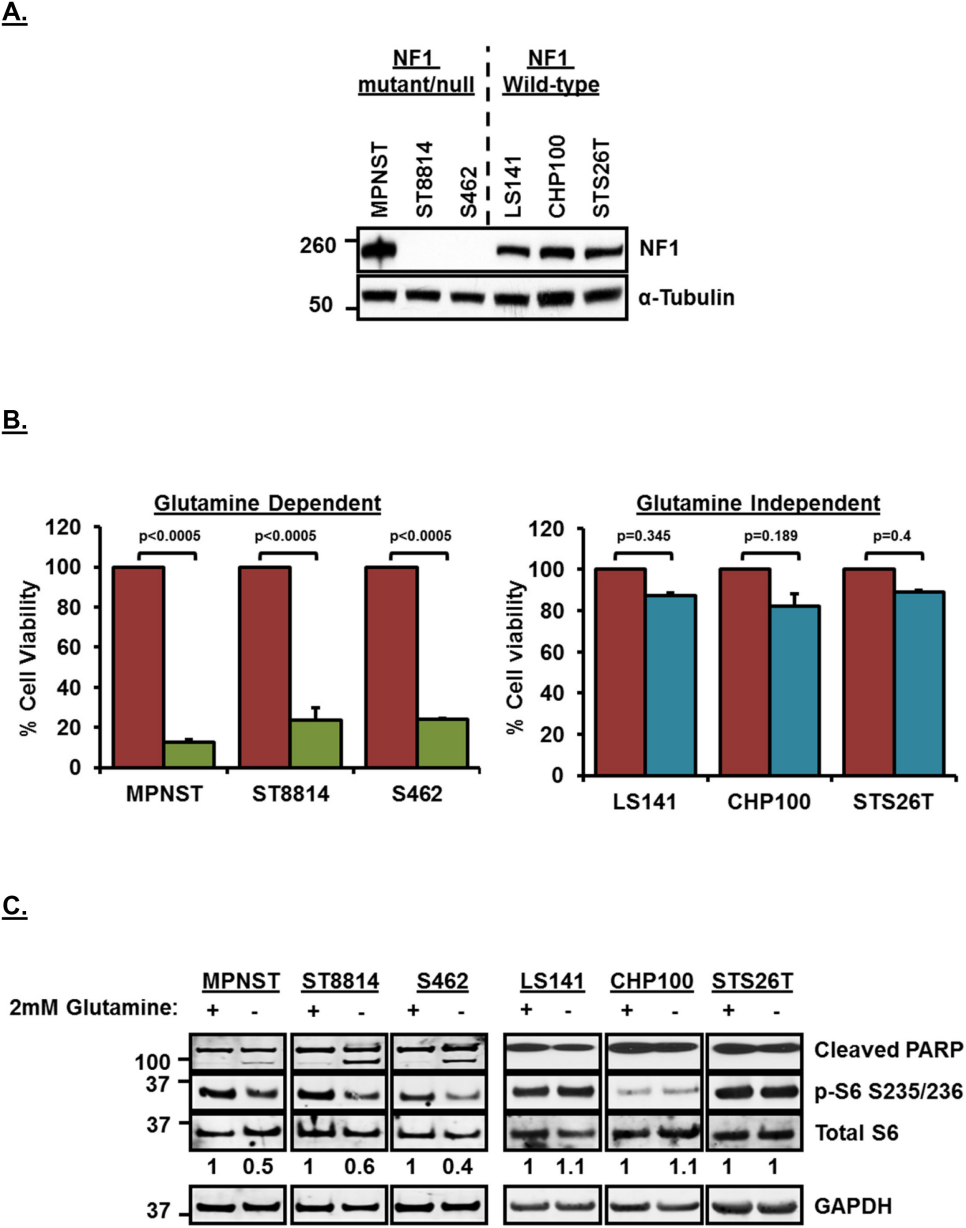
**(A)** NF1 expression levels in *NF1* mutant/null and *NF1* wild-type soft-tissue sarcoma cell lines. Cells from a confluent 60mm plate were washed twice with ice-cold PBS and cell pellet was obtained by scraping in PBS and centrifuging. Pellet was lysed with RIPA lysis buffer. 30μg of lysates were loaded on SDS/PAGE and proteins were detected on western blot using indicated antibodies. Numbers on the left indicate molecular weight in kilo Daltons (kDa). **(B)** Glutamine dependency of *NF1* mutant/null cell lines for cell proliferation.1500 cells per well were plated in 96 well plates in triplicate in RPMI+10%FBS without Glutamine for 24 hours. Next day, media was replaced with RPMI+10%FBS with or without 2mM Glutamine. After 72 hours, cell viability was measured using Dojindo CCK-8 kit using manufacturer’s instructions. Cell viability was calculated as percentage of growth in 2mM Glutamine containing media. Combined data from two independent experiments is shown. Error bars represent standard error mean. **(C)** Induction of apoptosis and downregulation of mTORC1 after glutamine deprivation in *NF1* mutant/null sarcoma cell lines. Cells were plated in RPMI+10%FBS without Glutamine for 24 hours. Next day, media was replaced with fresh RPMI+10%FBS without Glutamine or RPMI+10%FBS containing 2mM Glutamine. Cells were incubated for another 48 hours, harvested, cell pellets were lysed in RIPA lysis buffer and 30μg of lysates were loaded on SDS/PAGE. Proteins were detected on western blot using indicated antibodies. Numbers at the bottom of blot indicate densitometric quantitation of p-S6 signal normalized to total S6 levels. Numbers on the left indicate molecular weight in kilo Daltons (kDa). Representative blot from at least 3 independent experiments are shown.

Although *NF1* is known to play a role in the development of malignant peripheral nerve sheath tumors (MPNSTs), its role in modulating glutamine dependency has not been studied before. MPNST, ST8814 and S462 cell lines used in this study have been shown previously to carry a mutation/deletion in *NF1*, whereas, STS26T cell line carries wild-type *NF1* [18–20]. LS141 (Liposarcoma) and CHP100 (Ewing Sarcoma) cell lines, on the other hand, have been used extensively and both these cell lines have not been reported to harbor any *NF1* mutation/loss [19, 21–24] (also, personal communication with Kanojia D, Cancer Science Institute, Singapore). Figure 1A shows the expression levels of NF1 in the six soft-tissue sarcoma cell lines that were used in this study. MPNST cell line shows detectable levels of NF1 expression since it is *NF1* mutant, whereas, ST8814 and S462 cell lines do not show any detectable levels of NF1 on the western blot (Figure 1A).

To test the dependency of these cell lines on extracellular glutamine for cell proliferation, we carried out a cell viability assay in the presence or absence of glutamine (2mM) in the growth media. Removal of glutamine from the media significantly (p<0.0005) decreased cell viability (Figure 1B) only in the *NF1* mutant/null cell lines, MPNST, ST8814 and S462 compared to wild-type *NF1* sarcoma cell lines, LS141, CHP100 and STS26T. A similar decrease in cell viability was observed in an *NF1*-null metastatic melanoma cell line, MeWo [25] when glutamine was removed from the media, whereas, another melanoma cell line, 92.1 [26], that does not carry any *NF1* mutation/deletion did not show any decrease in cell viability after glutamine removal from the media (Supplementary Figure 1, left panel). This clearly suggested that cancer cell lines other than soft-tissue sarcoma carrying *NF1* mutation/deletion may also show sensitivity to glutamine deprivation. Western blot analysis shows that removal of glutamine from the media induced apoptosis (shown as induction of cleaved poly ADP-ribose polymerase, PARP) only in *NF1* mutant/null but not in wild-type *NF1* cell lines (Figure 1C and Supplementary Figure 1, right panel). Since glutaminolysis is known to play an important role in mTORC1 activation [27], removal of glutamine from media downregulated mTORC1 activity (shown as decreased phosphorylation of S6 ribosomal protein, p-S6 S235/236 and also quantitated in arbitrary densitometric units) in *NF1* mutant/null but not wild-type *NF1* cell lines (Figure 1C and Supplementary Figure 1).

### Glutaminase inhibition results in decreased cell proliferation, mTORC1 signaling and glutamine utilization in *NF1* mutant/null cell lines

**Figure 2:**
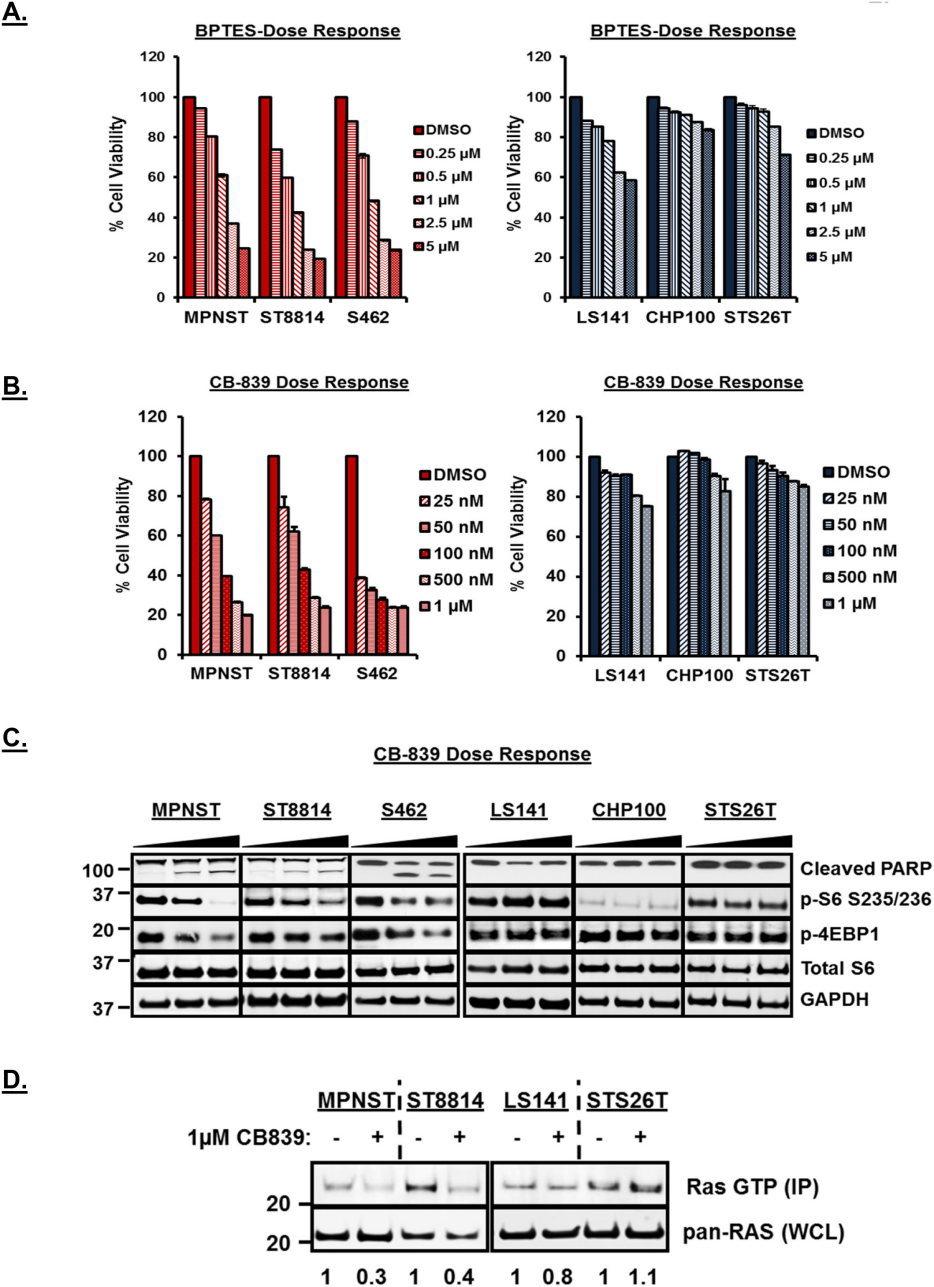
**(A** and **B)** Anti-proliferative efficacy of BPTES and CB-839 in *NF1* mutant/null (MPNST, ST88 and S462) versus *NF1* wild-type (LS141, CHP100 and STS26T) cell lines. 1500 cells per well were plated in 96 well plates in triplicate in RPMI+10%FBS without Glutamine for 24 hours. Next day, cells were treated with increasing dose of CB-839 or BPTES in RPMI+10%FBS with 2mM Glutamine. After 72 hours, cell viability was measured using Dojindo CCK-8 kit using manufacturer’s instructions. Cell viability was calculated as percentage of growth compared to DMSO control. Combined data from two independent experiments is shown. **(C)** Induction of apoptosis and downregulation of mTORC1 with the treatment of CB-839 in *NF1* mutant/null versus wild-type *NF1* cell lines. Cells were grown to 60% confluency in 60-mm plates for 24 hours and treated for another 48 hours with DMSO or increasing concentrations of CB-839 (0.1 and 1μM). 30μg of RIPA lysates were loaded on SDS/PAGE and immunoblotted using indicated antibodies. Representative blot from at least 3 independent experiments are shown. Numbers next to the blot indicate molecular weight in kilo Daltons (kDa). **(D)** Detection of active Ras in *NF1* mutant/null and *NF1* wild-type cell lines. *In vitro* Ras activity from cell lysates was determined using active Ras pull down and detection kit as described in materials and methods. Immunoprecipitated samples (IP) along with 25μg of total cell lysates (TCL) were analyzed by Western blotting using an anti-Ras mouse monoclonal antibody. Representative blot from at least 3 independent experiments are shown. Numbers next to the blot indicate molecular weight in kilo Daltons (kDa).

Inhibition of glutaminase, a key enzyme in the conversion of glutamine to glutamate, by BPTES [bis-2-(5-phenylacetamido-1,2,4-thiadiazol-2-yl)ethyl sulfide] and CB-839 has been shown to have anti-proliferative effect in triple negative breast cancer, leukemia and glioma [28–30]. To test the efficacy of glutaminase inhibition by BPTES and CB-839 in these cell lines, we carried out cell viability assays and western blot analysis using increasing concentrations of both the drugs. As shown in Figure 2A and 2B, both BPTES and CB-839 treatment resulted in a significant decrease (p<0.001) in cell viability only in *NF1* mutant/null cell lines (also Supplementary Figure 1, left panel). Notably, CB-839 treatment resulted in decreased cell viability at low nanomolar concentrations compared to BPTES (Figure 2A versus 2B). Western blot analysis (Figure 2C) after 48 hours of CB-839 treatment revealed induction of cleaved PARP as well as downregulation of mTORC1 targets such as p-S6 and p-4EBP1 in *NF1* mutant/null but not in wild-type *NF1* cell lines. Similar decrease in cell viability, induction of PARP and downregulation of downstream signaling pathways was observed for the *NF1*-null melanoma cell line, MeWo but not for the wild-type *NF1* carrying melanoma cell line, 92.1 (Supplementary Figure 1, right panel). CB-839 treatment at time points as early as 3 and 6 hours also showed downregulation of mTORC1 targets such as p-S6 only in the *NF1*-null cell line, ST8814 (Supplementary Figure 2).

To test whether the inhibitory effects observed after CB-839 treatment were solely a result of inhibition of mTORC1 activity by CB-839; we compared the effects of CB-839 treatment against a known mTORC1 inhibitor, rapamycin, in a cell proliferation and western blot analysis assay. Unlike CB-839, rapamycin treatment at 10nM [24] induced a modest inhibition of cell proliferation in the *NF1*-null (ST8814) cell line (Supplementary Figure 2). Complete inhibition of mTORC1 targets such as p-S6 was observed in both the cell lines (ST8814 and STS26T) irrespective of their *NF1*- status (Supplementary Figure 2). Only CB-839 but not rapamycin treatment induced cleaved PARP in the *NF1*-null ST8814 cell line (Supplementary Figure 2). No changes in the expression levels of GLS1 were observed after CB-839 or rapamycin treatment suggesting that the effects of CB-839 treatment are at the functional rather than expression levels of GLS1.

Neurofibromin (NF), a product of the *NF1* gene, is a RAS GTPase-activating protein and is well known to negatively regulate the RAS signaling pathway [31]. Since only the *NF1*- associated but not wild-type *NF1* cell lines were sensitive to glutaminase inhibition, we tested whether CB-839 treatment has any effect on Ras activity in these cell lines. To test this, we carried out an *in vitro* pulldown assay for active Ras (Ras GTP immunoprecipitated using Ras Binding Domain of Raf, RBD) using cell lysates from two *NF1* associated (MPNST, ST8814) and two wild-type *NF1* cell lines (LS141, STS26T) treated with 1μM CB-839 for 48 hours. Western blots in Figure 2D (top panel) show a significant decrease in immunoprecipitated Ras-GTP levels (active Ras) only in the *NF1*-associated but not wild-type *NF1* cell lines (also shown as arbitrary densitometric units). No decrease in total Ras was observed in any of the cell lines (Figure 2D, bottom panel). *NF1*-null melanoma cell line, MeWo, but not wild-type *NF1* carrying cell line 92.1, showed similar decrease in active Ras in response to CB-839 treatment (Supplementary Figure 3).

**Figure 3:**
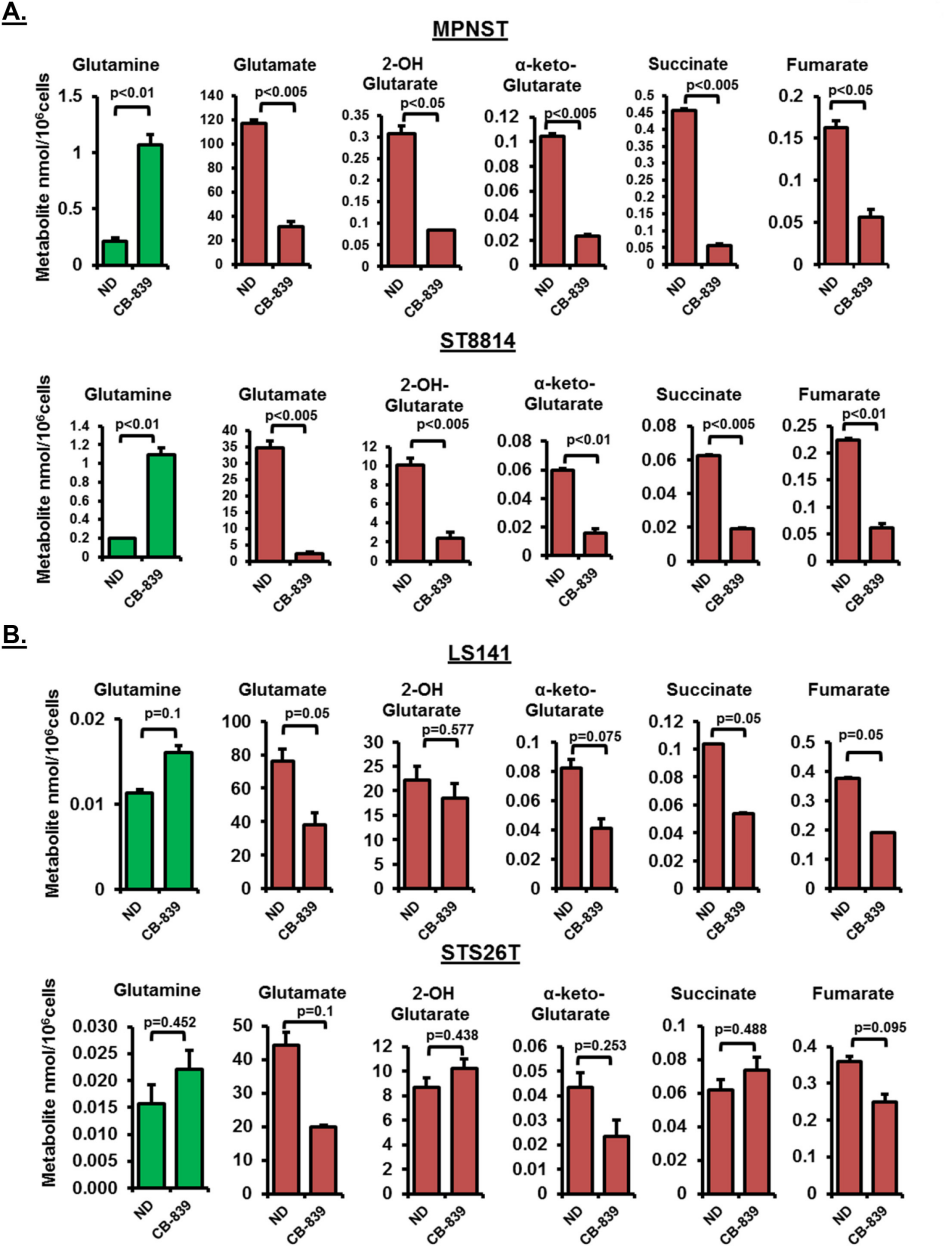
**(A** and **B)** Intracellular metabolite profiling after CB-839 treatment. Changes in intracellular metabolite levels in *NF1* mutant/null (MPNST, ST8814) and *NF1* wild-type (LS141, STS26T) cells treated with DMSO or 1μM CB-839 for 48 hours are shown. The levels of intermediary metabolites (nmol/10^6^cells) were determined by Gas chromatography-mass spectrometry (GC-MS) metabolomics as described in materials and methods. Results are representative of at least two independent experiments. Error bars represent standard error mean. ND=No Drug control.

In order to test the metabolic consequences of glutaminase inhibition, we carried out intracellular metabolite analysis in response to CB-839 treatment in two *NF1* associated (MPNST and ST8814) and two wild-type *NF1* cell lines (LS141 and STS26T). Treatment with 1μM CB-839 for 48 hours resulted in a significant decrease in glutamine utilization (p<0.01) shown as a steep increase in intracellular glutamine levels and a significant decrease in glutamate levels (p<0.005) (Figure 3A), thus, confirming reduced conversion of glutamine to glutamate. The increase in glutamine levels or the decrease in glutamate levels was not as significant in wild-type *NF1* cell lines (Figure 3A versus 3B). It must be noted that the basal metabolite levels for glutamine in the *NF1* associated cell lines were higher than the wild-type *NF1* cell lines (Figure 3A and 3B). In addition to glutamate levels, a decrease in other TCA intermediary metabolites such as succinate, fumarate and α-keto-Glutarate (α-KG) were also more pronounced in *NF1* associated compared to wild-type *NF1* cell lines (Figure 3A and 3B).

### Effects of glutamine deprivation can be restored by re-addition of glutamate to the media

**Figure 4:**
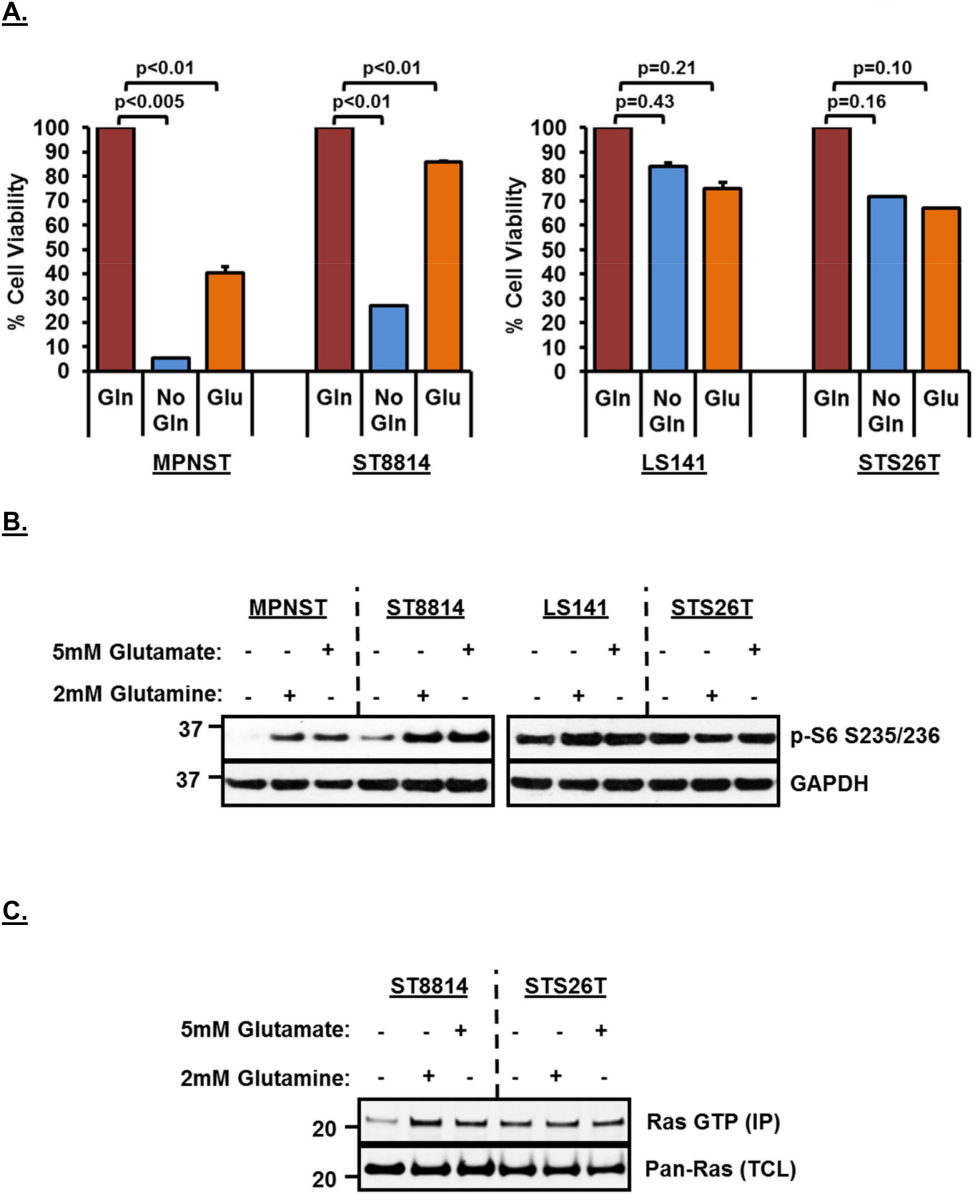
**(A)** Rescue of growth inhibition in *NF1* mutant/null cell lines by Glutamate supplementation in media. 1500 cells per well were plated in 96 well plates in triplicate in RPMI+10%FBS without Glutamine for 24 hours. Next day, media was replaced with fresh RPMI+10%FBS without Glutamine or RPMI+10%FBS containing 2mM Glutamine or 5mM Glutamate. After 72 hours, cell viability was measured using Dojindo CCK-8 kit using manufacturer’s instructions. Cell viability was calculated as percentage of growth in 2mM Glutamine containing media. Combined data from two independent experiments is shown. Error bars represent standard error mean. **(B** and **C)** Rescue of mTORC1 and Ras activity after re-addition of Glutamine or Glutamate in the media. Cells were plated in RPMI+10%FBS without Glutamine 24 hours. Next day, media was replaced with fresh RPMI+10%FBS without Glutamine or RPMI+10%FBS containing 2mM Glutamine or 5mM Glutamate. Cells were incubated for another 48 hours, harvested, cell pellets were lysed in RIPA lysis buffer and 30μg of lysates were loaded on SDS/PAGE. Proteins were detected on western blot using indicated antibodies. *In vitro* Ras activity from cell lysates was determined using active Ras pull down and detection kit as described in materials and methods. Representative blot from at least 2 independent experiments are shown. Numbers next to the blot indicate molecular weight in kilo Daltons (kDa).

In order to test whether the decreased cell viability is a result of reduced conversion of glutamine to glutamate, we carried out a rescue experiment by adding 5mM glutamate back to the media. Figure 4A shows that adding glutamate back to the media rescued cell viability in both *NF1* associated cell lines (more significantly in the *NF1* null ST8814 cell line among the two), thereby, strongly supporting the hypothesis that *NF1* associated cell lines are highly dependent on glutaminase activity for cell viability. Re-addition of either 2mM glutamine or 5mM glutamate to the growth media also resulted in restoring downregulated mTROC1 activity as indicated by increased p-S6 signal in *NF1* associated cell lines after re-addition (Figure 4B). As seen earlier (Figure 1C), wild-type *NF1* cell lines (LS141 and STS26T) did not show any decrease in mTORC1 signaling as a result of glutamine removal and therefore, p-S6 levels stayed the same and did not increase further despite re-addition of either glutamine or glutamate to the growth media (Figure 4B).

To test whether the decreased Ras activity in response to removal of glutamine from the media could also be restored, we carried our Ras pull-down assay after adding 2mM glutamine or 5mM glutamate back to the media. As shown in Figure 4C, re-addition of either glutamine or glutamate to the media restored active Ras (top panel) in the *NF1*-null cell line, ST8814. No change in active Ras was noted in the wild-type *NF1* cell line, STS26T, irrespective of the addition of glutamine or glutamate to the media. No decrease in total Ras was observed in any of the cell lines.

### Changes in NF1 expression levels modulates sensitivity and Ras activity in response to glutaminase inhibition

**Figure 5:**
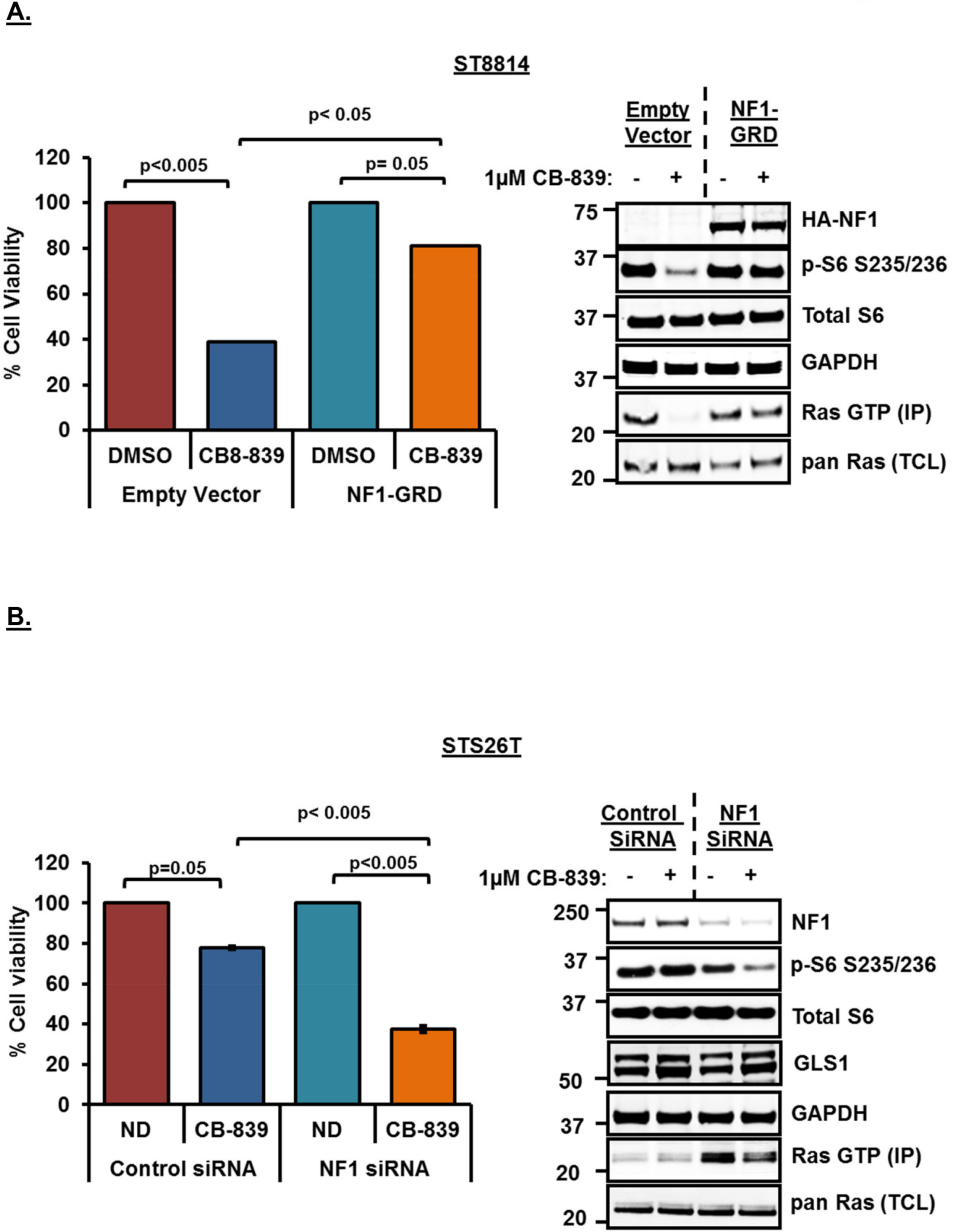
**(A)** Rescue of cell proliferation and mTORC1 activity by wild-type *NF1*-GRD overexpression. *NF1* null (ST8814) cells were plated overnight and transfected transiently with HA-tagged wild-type *NF1* GAP related domain (*NF1-GRD*) or an empty vector next day and incubated for another 48 hours. Cells were then trypsinized and 1500 cells per well were plated in 96 well plates in triplicate and 100 mm plates ( 10^6^ cells / plate) for cell viability and western blot analysis respectively. Cells were then treated next day with 1μM CB-839 or DMSO for another 72 hours. Cell viability was measured after using Dojindo CCK-8 kit using manufacturer’s instructions as described earlier. Cell viability was calculated as percentage of growth in DMSO control. Western blot analysis and active Ras pull down from the lysates was carried out as described in materials and methods. **(B)** siRNA mediated knockdown of wild-type *NF1* sensitizes STS26T cell line to glutaminase inhibition by CB-839. 100 nM siRNAs (pooled siRNAs, Dharmacon) specific for *NF1* or scrambled (non-targeting pool) siRNA as a control were transfected using Lipofectamine RNAiMAX reagent (Invitrogen). 48 hours after transfections, cells were trypsinized, counted using a Nexcelom cell counter and plated in 96-well plates in triplicate for cell viability assays or 60mm plates for western blotting analysis. Cells were also collected, lysed and analyzed by western blot to check for knockdown of protein expression. For western blotting, cells in 60mm plates were treated for 24 hours, whereas, for cell viability assays, cells in 96 well plates were treated for 72 hours with 1μM CB-839. Combined data from two independent experiments is shown. Numbers next to the blot indicate molecular weight in kilo Daltons (kDa).

In order to confirm the role of *NF1* in conferring sensitivity to glutaminase inhibition, we next carried out *NF1* overexpression as well as siRNA mediated *NF1* knockdown experiments. Overexpression of *NF1-GRD* (GAP Related Domain) has been shown previously to restore normal cell growth in *NF1*-/- cells [32] as well as downregulate high levels of Ras-GTP in leukemia cells [33]. To test this, we overexpressed wild-type *NF1-GRD* in the *NF1* null cell line, ST8814, and then treated the cells with 1μM CB-839 for 48 hours. As shown earlier, CB-839 treatment in empty vector transfected cells resulted in decreased cell viability and mTORC1 signaling (p-S6) as well as reduced Ras-GTP levels (Figure 5A). However, overexpression of *NF1-GRD* was able to rescue the effects of CB-839 treatment on cell viability as well as mTORC1 and active Ras (Figure 5A), thus, restoring p-S6 as well as Ras-GTP levels in NF1-overexpressing cells. Similar results were obtained in *NF1* null melanoma cell line, MeWo, (Supplementary Figure 3) indicating a strong correlation between *NF1* status and glutamine dependency.

We also carried out siRNA mediated knockdown of *NF1* in the wild-type *NF1* cell line, STS26T. As a result of *NF1* knockdown, STS26T cell line exhibited decreased cell viability, and reduced mTORC1 activity (p-S6) (Figure 5B). SiRNA mediated loss of *NF1* resulted in increased Ras-GTP levels (Figure 5B, western blot, lane 3). CB-839 treatment post *NF1* knockdown was able to reduce increased Ras-GTP levels (Figure 5B, western blot, lane 4) clearly suggesting glutamine dependency in these cells when *NF1* is knocked down. No changes in the expression levels of GLS1 were observed after SiRNA mediated knockdown of *NF1* or post CB-839 treatment (Figure 5B).

### Glutaminase inhibition by CB-839 in MPNST xenografts results in suppression of tumor volume and reduced glutamine utilization in tumors

**Figure 6:**
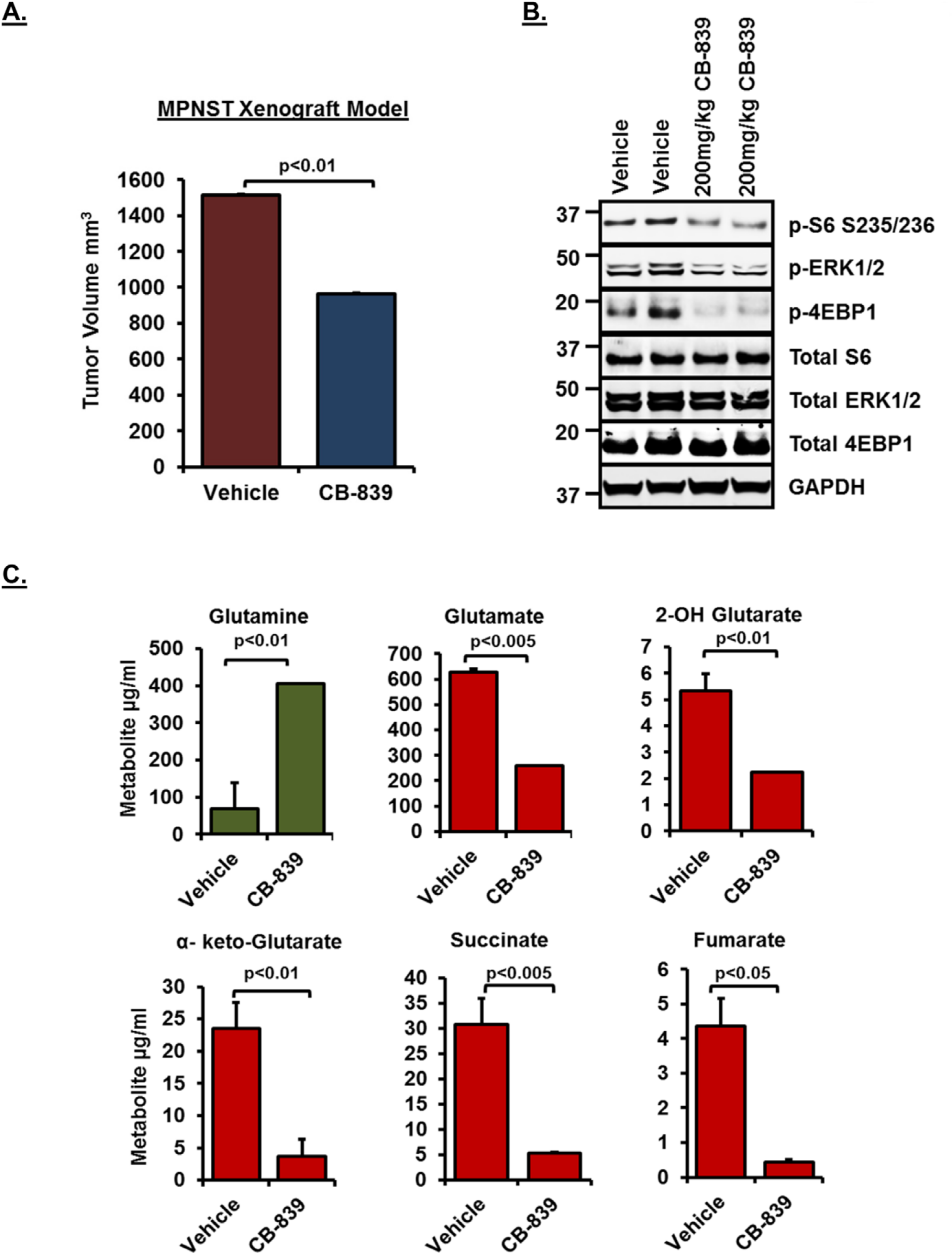
Effect of CB-839 treatment in MPNST xenografts **(A)** Tumor growth of MPNST xenografts treated with CB-839 or Vehicle control for a period of 2 weeks is shown. See methods for drug dosing schedule. **(B)** 30μg of RIPA lysates obtained using sample grinding kit (GE healthcare) from xenograft tissues at the end of 2-week treatment were loaded on SDS/PAGE and immunoblotted using indicated antibodies. Blots from tumor tissues obtained from two independent animals per treatment condition are shown. Numbers next to the blot indicate molecular weight in kilo Daltons (kDa). **(C)** Relative changes in intermediary metabolite levels (μg/ml) in MPNST xenografts treated with CB-839 or Vehicle controls (n=6) were measured by Gas chromatography-mass spectrometry (GC/MS) based metabolomics as described in materials and methods. Error bars represent standard error mean.

In order to test whether glutaminase inhibition by CB-839 can result in reduced tumor volume *in vivo*, we carried out mouse xenograft studies using MPNST serial transplant tumors [34]. As shown in Figure 6A, treatment with 200mg/kg CB-839 resulted in significant suppression of tumor volume compared to vehicle control (n=6). Western blot analysis (Figure 6B) revealed inhibition of downstream signaling pathways including p-ERK1/2 as well as mTORC1 targets such as p-S6 and p-4EBP1, thus, confirming the inhibition observed *in vitro*. Similar to the results obtained *in vitro*, metabolite profiling of tumor samples (Figure 6C) showed a significant decrease in glutamine utilization (shown as increased accumulation of glutamine in tumors) and reduced levels of TCA cycle intermediary metabolites such as α-keto-Glutarate, succinate and fumarate.

## DISCUSSION

The present study is the first report evaluating glutamine dependency and glutaminase inhibition as a potential therapeutic approach in *NF1* associated tumors. Though previous studies have evaluated efficacy of glutaminase inhibitors in cancers such as triple negative breast cancer [28], acute myeloid leukemia [35] and pancreatic cancer [36], glutamine dependency in *NF1* associated malignancies has not been studied before. Malignant peripheral nerve sheath tumors or MPNSTs arise in patients with type 1 neurofibromatosis (NF1) and are often associated with activation of the Ras pathway due to loss of function mutations in *NF1*, a gene which encodes the Ras-GTPase activating protein (GAP), Neurofibromin (NF) [37]. MPNSTs are often difficult to treat and the only definitive therapy despite recent advances relies on surgical resection of tumor. Though many molecularly targeted agents are currently being tested in clinical trials [38], the prognosis still remains poor and more effective clinical approaches are needed to treat MPNSTs. In addition to *NF1* associated MPNSTs, role of *NF1* mutations has also been elucidated in other cancer types such as cutaneous melanoma [25]. Although research aimed at understanding molecular mechanisms that drive *NF1* associated malignancies has been in focus for some time [13], not many treatment options are available to treat this disease and novel therapeutic approaches are urgently needed [39].

Cancer cells are known to utilize large amounts of glucose and glutamine in order to fuel the tricarboxylic acid (TCA) cycle. Glutamine, a critical nutrient for driving cancer cell proliferation, acts as a source of fatty acid, lipid as well as glutathione synthesis especially under hypoxic conditions [40]. Glutaminase, a key enzyme that converts glutamine to glutamate, is crucial for generation of intermediary metabolites such as α-keto-Glutarate that drive fatty acid production and glutathione synthesis [41]. Glutamine deprivation and glutaminase inhibition, therefore, has emerged as a potential therapeutic approach in recent years [11, 42–45]. Selective glutaminase inhibitors, such as CB-839 (N-(5-(4-(6-((2-(3-(Trifluoromethoxy)phenyl)acetyl)amino)-3-pyridazinyl)butyl)-1,3,4-thiadiazol-2-yl)-2-pyridineacetamide) have been the subject of clinical trials in solid tumors and hematological malignancies (ClinicalTrials.gov Identifier: NCT02071862, ClinicalTrials.gov Identifier: NCT02071888, and ClinicalTrials.gov Identifier: NCT02071927). Based on preclinical data, combination trials of CB-839 with EGFR inhibitors in EGFR mutant lung cancer and with proteasome inhibitors in multiple myeloma are now being planned [46, 47]. When we tested the effect of glutamine deprivation on cell viability in six soft-tissue sarcoma cell lines, we observed a significant decrease in cell viability and induction of apoptosis after glutamine removal from the media only in the *NF1* associated cell lines. This was surprising since no correlation has been reported previously between the *NF1* status and glutamine dependency. To explore further, we carried out cell viability and western blot analysis after glutaminase inhibition using either BPTES or CB-839 and observed that glutaminase inhibition not only decreased cell viability and induced apoptosis but also resulted in significant downregulation of p-S6, a known mTORC1 target. When we deprived the *NF1* mutant/null cells of glutamine in the media or treated the cells with glutaminase inhibitor, CB-839 and carried out *in vitro* detection of Ras activity, we observed a significant decrease in active Ras (Ras-GTP), thus, supporting our hypothesis that Ras activity in these cell lines is dependent on glutamine utilization. Moreover, this decrease in Ras activity could be recapitulated when *NF1* was knocked down using siRNA in the wild-type *NF1* cell line, STS26T. Conversely, when wild-type *NF1-GRD* (GAP Related Domain) was overexpressed in *NF1* null cell line (ST8814), the decrease in Ras activity in response to glutaminase inhibition could be restored. Taken together, our data strongly suggests that Ras activity in the *NF1* mutant/null cell lines is highly dependent on the ability of cells to utilize glutamine.

Interestingly, we observed that sensitivity to glutaminase inhibition is modulated depending on the presence or absence of wild-type *NF1*. Decreased cell viability in response to CB-839 treatment in *NF1* null cell line (ST8814) could be reversed when wild-type *NF1* was overexpressed. On the other hand, siRNA mediated knockdown of *NF1* in wild-type *NF1* cell line (STS26T) resulted in increased sensitivity to glutaminase inhibition.

In accordance with the previously reported studies [7, 27], we observed a reduction in mTORC1 signaling (shown as decreased p-S6 and p-4EBP1) in response to glutaminase inhibition and glutamine deprivation confirming the role played by glutaminolysis in mTORC1 activation. Similar to cell viability assays, knockdown or overexpression of *NF1* modulated mTORC1 activity in response to glutaminase inhibition. Thus, decreased mTORC1 (p-S6) signaling after CB-839 treatment was rescued when *NF1* was overexpressed, whereas, mTORC1 activity was reduced when *NF1* was knocked down.

Western blot analysis using tissue lysates obtained from tumor xenograft samples also showed a decrease in p-ERK as well as p-S6 and p-4EBP1, thus, validating the results obtained *in vitro*. Loss of *NF1* is known to induce Ras activation [31] and our data showing blockade of p-ERK (a critical component of Ras/Raf/ERK pathway) in response to glutaminase inhibition suggested that glutamine metabolism in the *NF1* associated tumors is important for Ras activity.

Decreased glutamine utilization after CB-839 treatment has been reported earlier in triple negative breast cancer [28]. Intermediary metabolite profiling showed a similar decrease in utilization of glutamine and a significant reduction in key TCA metabolites such as α-keto-Glutarate, succinate and fumarate in *NF1* associated cell lines (MPNST and ST8814) compared to wild-type *NF1* cell lines (LS141 and STS26T) as well as in MPNST tumor xenograft samples *in vivo*. It was noteworthy that cell viability as well as mTORC1 signaling and Ras activity were rescued when one of the key metabolites, glutamate, was provided in the media suggesting that cells were able to take up externally added glutamate, thus, overcoming the blockade of intracellular glutamine utilization. It must be noted though that the glutamate levels were similar in both glutamine dependent and independent cell lines despite intracellular levels of glutamine being low in glutamine independent cell lines. We postulate that these cell lines are able to generate intermediary metabolites like glutamate via glucose consumption since no glutamine dependency was observed. On the other hand, cell lines highly dependent on glutamine (MPNST and ST8814) are able to generate glutamate mostly via glutaminolysis. Therefore, the decrease in glutamate levels post CB-839 treatment is more pronounced in glutamine dependent cell lines compared to glutamine independent cell lines. More studies need to be undertaken to understand the intricacies of glutamine versus glucose consumption in *NF1* mutant/null and *NF1* wild-type cell lines. The other notable observation from the metabolite profiling analysis was the high-levels of the intermediary metabolite 2-hydorxy-glutarate (2-HG), a known oncometabolite, in the *NF1*-null ST8814 as well as wild-type *NF1* carrying LS141 and STS26T cell lines. Cancer associated mutations in isocitrate dehydrogenase (IDH) often lead to high levels of 2-HG [48]. While the D-isomer of 2-HG is often associated with mutant IDH, L-isomer is produced under hypoxic conditions [49]. The metabolites quantitated by the mass spectrometry approach used in this study are L-enantiomers. Moreover, previous mutational genomic analysis of the *NF1*-null ST8814 [20] and wild-type *NF1* carrying LS141 [22] cell lines have not identified any mutations in IDH, therefore, we do not anticipate that the high levels of 2-HG observed in these cell lines are a result of possible IDH mutations.

Glutamine has been shown to play a major role not only as a carbon and nitrogen source but also contributes to the anti-oxidative pathways and chromatin organization [50]. Other studies have shown that pancreatic cancer cells with oncogenic KRas are dependent on glutamine metabolism for proliferation [51]. As mentioned earlier, studies have reported that mTORC1 activation is dependent on glutaminolysis and glutamine metabolism is required to promote cell growth and proliferation [27]. We believe that in our study, *NF1* mutant/null cells are highly dependent on glutamine metabolism for proliferation and withdrawal of glutamine from media or inhibition of GLS1 by CB-839 results in decreased glutaminolysis and thus, reduced mTORC1 activity (shown as reduced p-S6 and p-4EBP1). Additionally, we observe that the Ras activity in our *NF1* mutant/null cells is highly dependent on glutamine metabolism. Therefore, we believe that the decreased cell proliferation in the *NF1* mutant/null cells is a combined result of decreased mTORC1 and Ras activity and this can be rescued either by overexpression of *NF1*-GRD or exogenous addition of glutamate to the media.

In summary, our data shows a strong correlation between *NF1* status and glutamine dependency in tumors. In addition to MPNSTs, a known *NF1* associated malignancy, such a correlation was also observed only in *NF1* null metastatic melanoma cell line, MeWo but not wild-type *NF1* carrying 92.1 cell line. This observation certainly warrants further studies to validate whether observed glutamine dependency is a universal phenomenon in other *NF1* associated malignancies such as leukemia and gliomas. Nonetheless, data from our study provides a strong rationale to explore targeted glutaminase inhibition as a potential therapeutic approach in the *NF1* disease setting. In fact based on these results a single agent CB-839 study for patients with *NF1* mutant MPNST, as well as a “basket” study for all *NF1* mutant patients, are now planned.

## MATERIALS AND METHODS

### Chemicals and drugs

CB-839 was provided by Calithera Biosciences. BPTES was purchased from Sigma Aldrich. Both the drugs were dissolved in dimethyl sulfoxide (DMSO) for *in vitro* studies and stored at -20°C.

### Cell culture and reagents

*NF1* null ST8814 and S462 cell lines were a kind gift by Dr. Karen Cichowski (BWH Biomedical Research Institute, Boston, MA). *NF1* mutant MPNST cell line has been described elsewhere [18]. STS26T, a wild-type *NF1* harboring MPNST cell line, was a generous gift by Dr. Steven. A Porcelli (Albert Einstein College of Medicine, Bronx, NY). De-differentiated Liposarcoma cell line (LS141) was obtained from Dr. Samuel Singer (Memorial Sloan Kettering Cancer Center (MSKCC), New York, NY). Ewing Sarcoma (CHP100) cell line was obtained from Dr. Melinda S. Merchant (Center for Cancer Research, NCI/NIH, Bethesda, MD). MeWo cell line was purchased from American Type Culture Collection All the cell lines were cultured in RPMI media with 10% FBS, 100 U/mL penicillin, and 100 mg/mL streptomycin, maintained at 37°C in 5% CO2, and passaged for no more than 4 months. Initial stocks of all cell lines were received from their sources within the past 3 years. Cell lines were determined to be mycoplasma free using MycoAlert Mycoplasma detection Kit (Lonza).

### Cell viability assays

For Glutamine depletion experiments, cells were plated in 96-well plates at 1,500-2,000 cells per well density in 0.1 ml Glutamine free RPMI media, and then treated with the indicated drugs in RPMI containing 2mMol/L Glutamine the next day for an additional 72 hours. Cell viability assays were carried out using the Dojindo Molecular Technologies (CCK-8) kit as per manufacturer’s instructions. The optical density was read at 450nm using a Spectra Max 340 PC (Molecular Devices Corporation). Cell viability is expressed as a percentage of untreated cells. Half maximal inhibitory concentrations (IC_50_) were extrapolated from cell viability data using CompuSyn software according to the manufacturer’s instructions.

### Western immunoblotting

Cells were lysed with 1X radioimmunoprecipitation assay (RIPA) buffer (Cell Signaling Technologies) supplemented with protease inhibitor cocktail tablets (Roche Diagnostics) and 1mM Na3VO4 (Sodium orthovanadate). Equal amounts (20-30μg) of protein were electrophoresed onto 4% to 12% gradient gels (Life Technologies) and transferred onto polyvinylidene difluoride (PVDF) membrane (Immobilon-FL, catalog # IPFL00010). Membranes were blocked in 1X Tris-buffered saline (TBS) containing 5% non-fat dry milk and 0.1% Tween 20 (TBS-T), and probed with primary antibodies. A complete list of antibodies used in the study is included in the supplementary methods. Bound antibodies were detected with horseradish peroxidase secondary antibodies (GE Healthcare) and visualized by Enhanced Chemiluminescence Reagent (GE Healthcare).

Tissues from xenograft experiment were homogenized using a mortar and pestle. Lysates were prepared using sample grinding kit form GE-healthcare (catalog # 80-6483-37), lysed with radioimmunoprecipitation assay (RIPA) buffer and western blotting was carried out as described above.

### Quantitation of organic acids by GC-MS

Standards for organic acids (Succinic acid, fumaric acid, alpha keto glutaric acid, citric acid, 2 hydroxyglutaric acid and amino acids (asparagine, aspartic acid, glutamic acid and glutamine) were purchased from Sigma (St. Louis, MO). Internal standard, succinate d4 was obtained from CDN Isotopes, Quebec, Canada. N, O Bis (trimethylsilyl) trifluoroacetamide (BSTFA) + 1% Trimethylsilyl chloride (TMCS) were purchased from Regis Technologies (Morton Grove, IL). Mox reagent (methoxamine in pyridine) was purchased form Supelco (St. Louis, MO). All other chemicals were of analytical reagent grade.

Cells were plated at a density of 3000 cells per plate in 60mm plates (Corning) in Glutamine free RPMI 1640 media supplemented with 10% FBS. 18 h after seeding, cells were treated with indicated drug in RPMI containing 2mM Glutamine. After 48 hours, culture medium was aspirated and attached cells were washed twice with cold saline water (9 gm/L NaCl) and quenched with 1.5ml ice-cold methanol. After 5 minutes of incubation on ice, cells were collected by scraping with disposable cell scraper (Fisher Scientific) and collected in glass tubes with Teflon sealed caps (Corning Inc. catalog # 9826-16X). One volume of chloroform was added to the cell suspension and vortexed at 4°C for 10 seconds. 1.5 ml water was added and vortexed for another 1 minute. Samples were centrifuged at 2000 rpm at 4°C for 20 min and the aqueous phase was collected in a new tube and evaporated under airflow at 37°C with nitrogen using an evaporator (Turbovap).

The extracted samples were evaporated under nitrogen stream prior to derivatization. The derivatization involves the addition of the internal standard, succinate d4 followed by 50uL of methoxyamine-HCl in pyridine and incubating for 90 min at 37°C. The final derivatization step involves the addition of 80uL of BSTFA+1% TMCS and incubating at 60°C for 30min.

### GC-MS analysis

GCMS analysis was performed using an Agilent 7000 triple quadrupole mass spectrometer coupled to an Agilent 7693 GC with an autosampler (Agilent Technologies, Palo Alto, CA).

Two microliters of the sample was injected onto a GC column (30mx0.2mmx0.25um) at a flow rate of 1mL/min. The initial temperature of the oven was set to 70°C kept for 1min followed by a ramp of 5°C/min to 200°C, held for 1 min and increasing to 300°C at 40°C/min with a total run time of 33.5min. The compounds were scanned in EI and spilt less injection mode by selected ion monitoring (SIM) using the following diagnostic ions: succinate, m/z 247; fumarate, m/z 245; alpha ketoglutarate, m/z 288; 2 hydroxyglutaric acid, m/z 247; citrate, m/z 273; glutamate, m/z 246; aspartic acid, m/z 232; asparagine m/z 231; glutamine, m/z 245; and succinate d4, m/z 251. Data acquisition, peak integration and quantitation were done by Mass Hunter software (version B. 07.04). The intra-assay precision was in the range of 1.5-6.0% and the accuracy was between 92-106%. The inter-assay precision and accuracy was in the range of 4.0-9.1% and 92-110% respectively.

### Gene silencing

Cells were plated at 50–60% confluency in 60 mm plates (Corning) and incubated for 24 h. Cells were then transiently transfected using Lipofectamine RNAiMAX (Invitrogen) using pooled siRNAs (GE Dharmacon). Nonspecific scrambled siRNA was used as control. After 48 hours of transfection, cells were lysed for western immunoblotting analysis.

### Detection of Ras activity

To determine the activity of Ras, EZ-Detect Ras Activation Kit (catalog # 89855, Thermo Scientific Pierce, Rockford, IL) was used according to manufacturer’s protocol. Briefly, cells were plated in RPMI+10%FBS without Glutamine and grown to approximately 60% confluency. Next day, media was replaced with fresh RPMI+10%FBS either without Glutamine or RPMI+10%FBS containing indicated reagents and incubated for another 48 hours. Cell lysates were prepared using the lysis buffer provided in the kit and then treated with GTPγS or GDP to activate or inactivate Ras, respectively. The nucleotide exchange reaction was terminated within 15 minutes by placing the samples on ice. The lysates were then incubated with a GST-fusion protein containing the Ras Binding Domain (RBD) of Raf1 to pull down active Ras. Detection of Ras was done by immunoblotting using pan-Ras antibody provided with the kit

### Xenograft studies

Briefly, MPNST xenografts were transplanted subcutaneously in the flank of ICR/SCID mice. Once tumors reached a volume of 80–100mm3, the mice were randomized into two groups of 7–10 animals each and treated with vehicle control or CB-839 (200mg/kg/day) orally for 2 weeks. Vehicle and CB-839 were prepared as described previously [28]. Tumor size was measured twice weekly by caliper. The average tumor volume in each group was expressed in cubic millimeter and calculated using the formula p/6 × (large diameter) × (small diameter) [2]. Animals were sacrificed after 3 weeks of drug treatment and the resected tumor tissues were snap frozen for western blot analysis. Experiments were carried out under an Institutional Animal Care and Use Committee-approved protocol, and institutional guidelines for the proper and humane use of animals were followed.

### Statistical analysis

All *in vitro* experiments were carried out at least three times unless otherwise indicated. P-values were calculated using Student’s T-test, with values of ≤0.05 determined to be statistically significant. Standard error was calculated as the standard deviation divided by the square root of the number of samples tested.

## SUPPLEMENTARY MATERIALS FIGURES


