# Supplementary material for: Targeted inhibition of glutaminase as a potential new approach for the treatment of NF1 associated soft tissue malignancies
Source: Oncotarget. 2017 Oct 6;8(55):94054–68. doi: 10.18632/oncotarget.21573 (PMC5706855; doi:10.18632/oncotarget.21573)
Supplement: Supplementary file 1 [file oncotarget-08-94054-s001.pdf]

Targeted inhibition of glutaminase as a potential new approach for the treatment of *NF1* associated soft tissue malignancies

SUPPLEMENTARY MATERIALS

A.

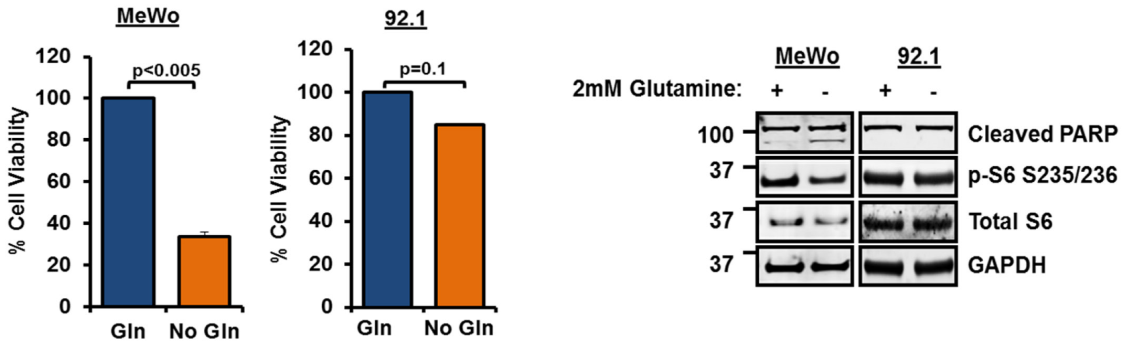

B.

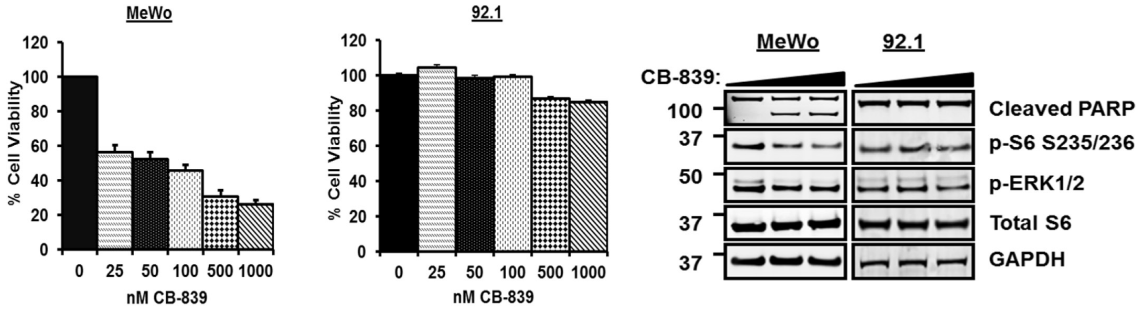

Supplementary Figure 1: (A and B). Glutamine dependency and effect of CB-839 in NF1 null MeWo versus wild-type NF1 carrying 92.1 melanoma cell line. Cell viability assay and western blot analysis after glutamine deprivation or CB-839 treatment was carried out as described in Figures 1 and 2.

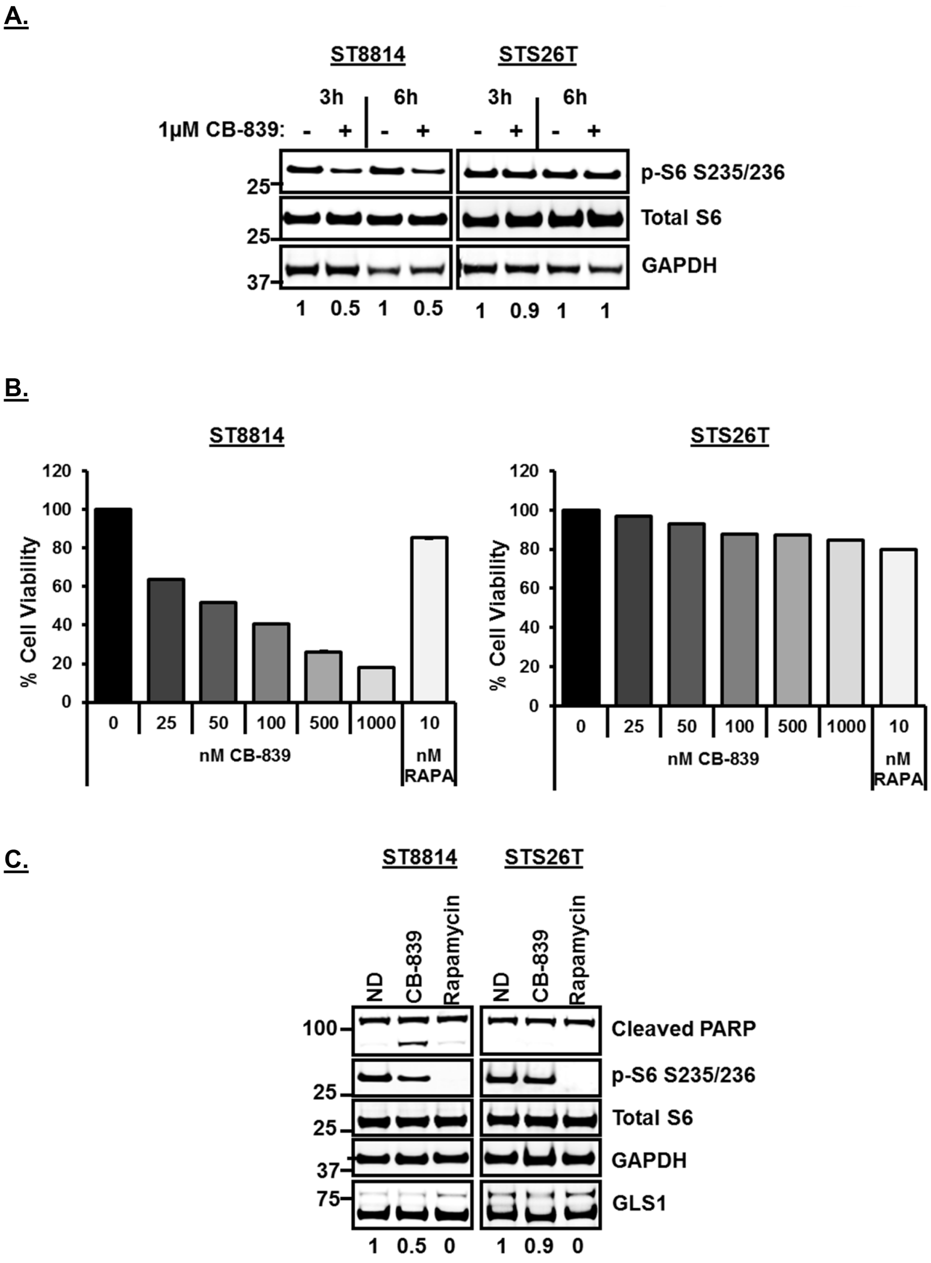

Supplementary Figure 2: (A). Downregulation of mTORC1 target p-S6 after 3h and 6h of CB-839 treatment in NF1 null (ST8814) versus wild-type NF1 cell line (STS26T). Cells were grown to 60% confluency in 60-mm plates for 24 hours and treated with DMSO or 1µM CB-839 for the indicated time points and harvested for western blot analysis. Western blot analysis was carried out as described in Figures 1 and 2. **(B).** Anti-proliferative efficacy of CB-839 versus Rapamycin in *NF1* null (ST8814) versus *NF1* wild-type (STS26T) cell line. 1000-2000 cells per well were plated in 96 well plates in triplicate in RPMI+10%FBS without Glutamine for 24 hours. Next day, cells were treated with increasing dose of CB-839 or 10nM Rapamycin in RPMI+10%FBS with 2mM Glutamine. After 72 hours, cell viability was measured using Dojindo CCK-8 kit using manufacturer's instructions. Cell viability was calculated as percentage of growth compared to DMSO control. **(C).** Induction of apoptosis and downregulation of mTORC1 target p-S6 with the treatment of CB-839 and Rapamycin in *NF1* null versus wild-type *NF1* cell lines. Cells were grown to 60% confluency in 60-mm plates for 24 hours and treated with DMSO, 1µM CB-839 or 10nM Rapamycin for another 48 hours, harvested cell pellets were lysed in RIPA lysis buffer and 30µg of lysates were loaded on SDS/PAGE. Proteins were detected on western blot using indicated antibodies.

A.

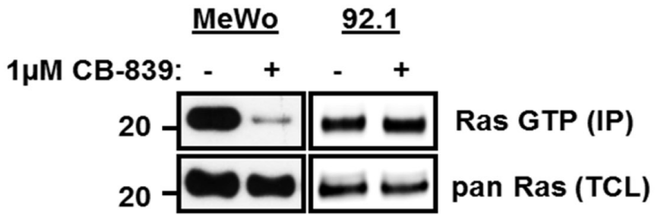

B.

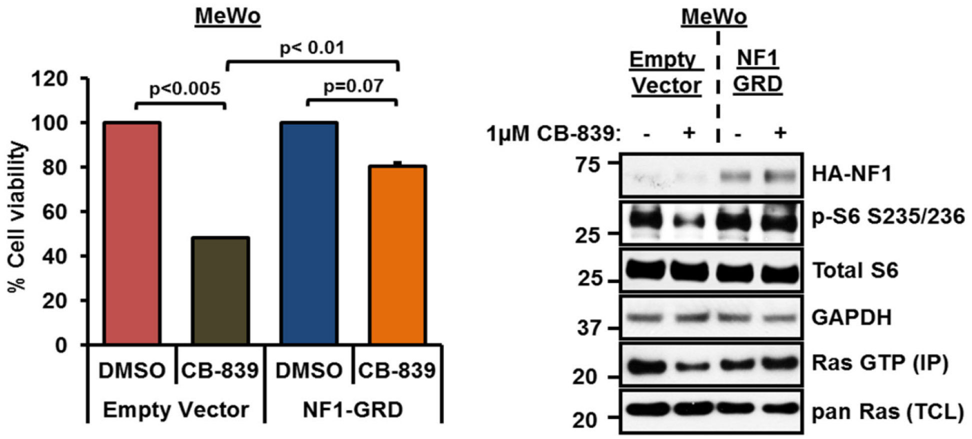

Supplementary Figure 3: (A). Detection of active Ras (Ras-GTP) in *NF1* null and *NF1* wild-type cell lines. **(B).** Rescue of cell proliferation and mTORC1 activity by wild-type *NF1*-GRD overexpression in metastatic melanoma MeWo cell line. Active Ras pull down as well as cell viability assay and western blot analysis after NF1-GRD overexpression was carried out as described in Figure 5.
